# Polyurethane Composites Reinforced with Walnut Shell Filler Treated with Perlite, Montmorillonite and Halloysite

**DOI:** 10.3390/ijms22147304

**Published:** 2021-07-07

**Authors:** Sylwia Członka, Agnė Kairytė, Karolina Miedzińska, Anna Strąkowska

**Affiliations:** 1 Institute of Polymer & Dye Technology, Lodz University of Technology, 90-924 Lodz, Poland; karolina.miedzinska@dokt.p.lodz.pl (K.M.); anna.strakowska@p.lodz.pl (A.S.); 2 Laboratory of Thermal Insulating Materials and Acoustics, Faculty of Civil Engineering, Institute of Building Materials, Vilnius Gediminas Technical University, Linkmenu St. 28, LT-08217 Vilnius, Lithuania; agne.kayrite@vilniustech.lt

**Keywords:** polyurethane composites, walnut shells, perlite, montmorillonite, halloysite, high-energy ball milling process

## Abstract

In the following study, polyurethane (PUR) composites were modified with 2 wt.% of walnut shell filler modified with selected mineral compounds–perlite, montmorillonite, and halloysite. The impact of modified walnut shell fillers on selected properties of PUR composites, such as rheological properties (dynamic viscosity, foaming behavior), mechanical properties (compressive strength, flexural strength, impact strength), dynamic-mechanical behavior (glass transition temperature, storage modulus), insulation properties (thermal conductivity), thermal characteristic (temperature of thermal decomposition stages), and flame retardant properties (e.g., ignition time, limiting oxygen index, heat peak release) was investigated. Among all modified types of PUR composites, the greatest improvement was observed for PUR composites filled with walnut shell filler functionalized with halloysite. For example, on the addition of such modified walnut shell filler, the compressive strength was enhanced by ~13%, flexural strength by ~12%, and impact strength by ~14%. Due to the functionalization of walnut shell filler with thermally stable flame retardant compounds, such modified PUR composites were characterized by higher temperatures of thermal decomposition. Most importantly, PUR composites filled with flame retardant compounds exhibited improved flame resistance characteristics-in all cases, the value of peak heat release was reduced by ~12%, while the value of total smoke release was reduced by ~23%.

## 1. Introduction

Polyurethanes (PUR) were first synthesized by Wurtz in 1849. A few decades later, in 1937, Otto Bayer obtained polyurethane in the known to this day, polyaddition reaction of polyol and polyisocyanate [1]. Now polyurethanes are widely used in different applications, such as building construction, packaging, and furnishing [2,3]. Polyurethanes are composed of rigid (hydrogen bonds) and flexible (the rest of the polyol chain) segments. After the formation of polyurethane macromolecule, the rigid segments are joined together, which leads to the formation of soft and hard domains [4]. The polyaddition reaction leading to the production of polyurethane materials is carried out in the presence of chain extenders, catalysts, flame retardants, and blowing agents [5]. Due to the wide range of selection and modification of used raw materials, polyurethane products can be obtained in various forms, including foams, coatings, sealants, adhesives, films, and fibers [6,7,8].

Foams have the largest share in the polyurethane materials market. PUR foams are divided into rigid, semi-rigid, and flexible.

Rigid foams are mainly used in building construction, to fill empty spaces near the window and door frames, for thermal and acoustic insulation [9,10]. Polyurethanes decompose at temperatures about 200 °C [11]. PUR materials are flammable, and their combustion process is accompanied by the release of heat and toxic gases. The easy ignition and high flame spreadability of polyurethane foams are the main disadvantages that noticeably limiting of PUR foams in many applications [12]. However, it is possible to use special additives aimed at reducing the flammability of the obtained materials–flame retardants. In line with the environmental goals and the principles of Sustainable Development, in recent years the research has been conducted to search for more and more natural and effective modifiers for polymer materials. To move away from chlorine-containing flame retardants, so-called non-halogen substances based on nitrogen, phosphorus, silicon, and boron compounds are used [13,14]. Moreover, recently more modifiers, not only flame retardant, have been used. Fillers of natural and plant origin may also positively affect the mechanical properties of the obtained composites. The literature commonly describes the use of natural additives in polymeric materials [8,9,15,16,17,18,19,20].

Perlite (P) is an inorganic chemically inert in many environments compound, composed mainly of SiO_2_, Al_2_O_3_, Na_2_O, K_2_O, and water [21]. Accordingly, with the estimation of global production, the leading producers of world production of perlite are China (47%), Greece (20%), Turkey (16%), and United States (13%) [22]. Perlite is an amorphous volcanic glass showing very low density and high porosity. It is also characterized by high thermal stability. Interestingly, when heated to a temperature between 760 and 1100 °C, perlite can expand even 7–16 times its original volume and acquires the properties of a thermal and acoustic insulator [23,24]. Perlite exhibits low thermal conductivity and good fire resistance, which makes it a potentially good flame retardant modifier [25].

Another interesting inorganic compound is montmorillonite (MMT), described with the chemical composition of Al_2_O_3_·4SiO_2_·3H_2_O. Montmorillonite has a layered structure, where the octahedron Al-O layer is between two tetrahedron Si-O layers [26,27]. MMT is characterized by a large specific surface, due to this it may delay thermal degradation and create a thermal barrier [28]. Due to its properties, in recent years montmorillonite has been increasingly used to increase the fire retardancy and thermal stability of polymer composites [29]. It may also improve the mechanical properties of the obtained materials which additionally increases its attractiveness as a modifier [30].

Halloysite is another layered mineral. It is aluminosilicate clay with the chemical composition of Al_2_Si_2_O_5_(OH)_4_. Naturally, halloysite occurs as small cylinders. It is characterized by a layered structure and specific surface area [31]. When heated to high temperatures, the halloysite loses the water between layers. Halloysite can be used as reinforcement in the preparation of polymer materials due to its physico-chemical properties and thermal stability [32,33]. Recently, it was proved that the layered structure of the halloysite can alter the thermal stability and flammability properties of polymer composites [34,35,36].

In the present study, the influence of modified walnut fillers on mechanical properties and the burning behavior of rigid polyurethane foams was determined. In our previous studies [37,38,39], the effect of polyol obtained based on walnut shells and silane-modified walnut filler on the properties of PUR foams. However, to our best knowledge, there have been no studies related to the modification of walnut fillers in the direction of reducing flammability. A goal of this study was to investigate the influence of non-halogen fire retardants (perlite, montmorillonite, and halloysite) as modifiers of walnut fillers.

## 2. Results and Discussion

### 2.1. Filler Characterization

**Figure 1:**
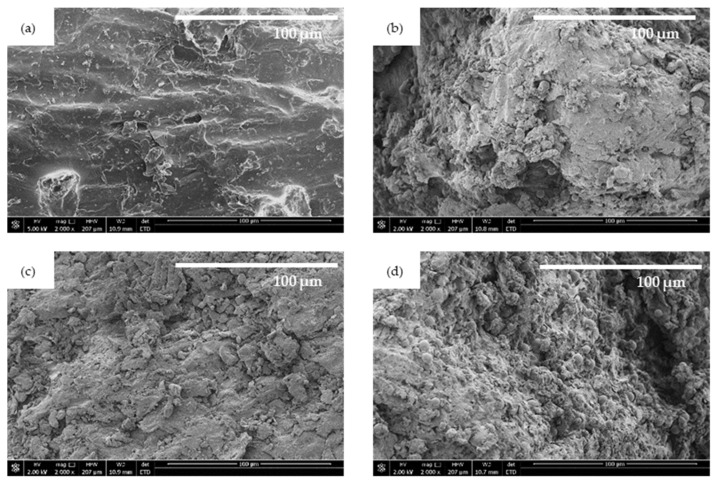
The external surface of (**a**) unmodified walnut shell filler, and walnut shell filler modified with (**b**) perlite, (**c**) montmorillonite, and (**d**) halloysite.

The structure of walnut shell fillers was examined using scanning electron microscopy (SEM). The obtained images (Figure 1) revealed that before the treatment, the surface of walnut shell filler is quite smooth and uniform. After the modification of walnut shell filler with perlite, montmorillonite, and halloysite the external morphology of the fillers seems to be less uniform with visible particles of the modifying compounds located on the surface of the walnut shell filler. This confirms a successful modification of walnut shell filler with perlite, montmorillonite, and halloysite.

**Figure 2:**
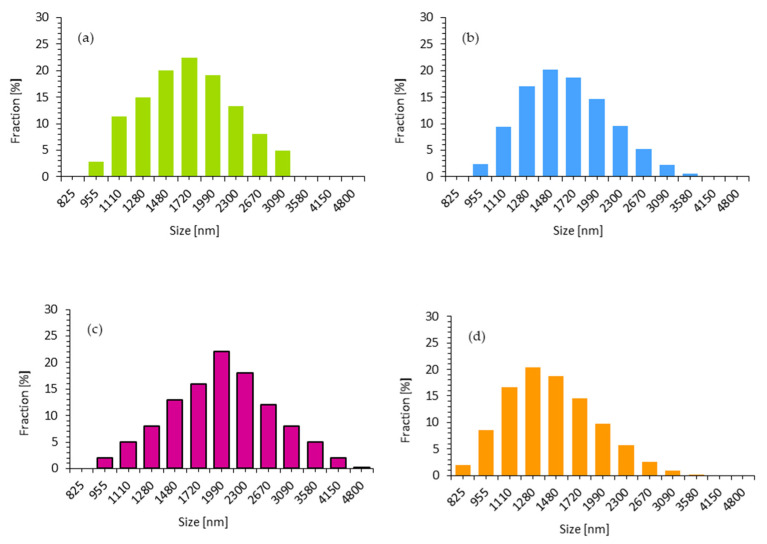
Particle size distribution of (**a**) WS (**b**) WS/P, (**c**) WS/MMT, and (**d**) WS/HL.

This, in turn, determines the further properties of PUR foams. The size of walnut shell fillers modified with perlite, montmorillonite, and halloysite was measured in a polyol dispersion. According to the results presented in Figure 2, the size of walnut shell fillers ranges between 900 and 4000 nm. In the case of unmodified walnut shell filler, the highest percentage (22.5%) is shown by the particles with an average size of 1720 nm. After the modification of the walnut shells with perlite and halloysite the average size of the particles decreases to 1480 nm (20.2% of the particles) and 1280 (20.4% of the particles), respectively. The particles of walnut shell filler modified with montmorillonite exhibit an average size of 1990 nm (22.5% of the particles).

**Figure 3:**
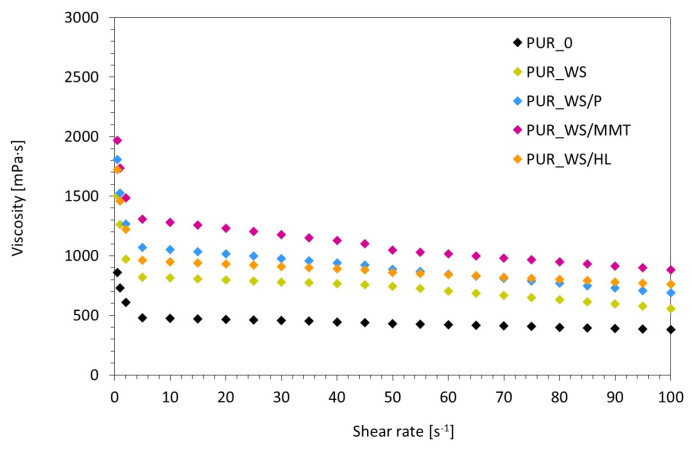
The results of dynamic viscosity of PUR systems containing WS fillers.

The viscosity of the PUR systems is a critical parameter, which affects the foaming process and the proper expansion of the cells [40,41]. According to the results presented in Figure 3, the addition of walnut shell fillers affects the viscosity of the PUR systems. When compared with the reference PUR system (without the addition of the filler), the PUR systems containing each type of walnut shell fillers exhibit an increased viscosity due to the presence of walnut shell filler particles. This may be connected with fact that the filler particles can interact with the polyether polyol through physical interactions—mainly hydrogen bonding and van der Wall interaction, increasing the total viscosity of the PUR system [42]. Compared to the reference PUR system, the greatest increase in dynamic viscosity exhibits PUR system with the addition of walnut shell filler modified with montmorillonite—the dynamic viscosity (measured at 0.5 RPM) increases from 860 to 1950 mPa·s. Most importantly, in the case of each PUR system, the viscosity decreases with increasing the shear rates. Such behavior is characteristic for non-Newtonian fluids with a pseudoplastic nature [43,44].

### 2.2. Characterization of PUR Composites Reinforced with Walnut Shell Fillers

The foaming behavior was evaluated by measuring the processing times of the PUR synthesis—start, growth, and tack-free times. The start time was measured from the start of mixing of components to a visible start of foam growth, extension time elapsing until reaching the highest volume of the foam, and gelation time was determined as the time when the foam solidifies completely and the surface is no longer tacky [45]. According to the results presented in Table 1 the addition of walnut shell fillers affects the value of start time and growth time. When compared with reference PUR foam, the value of start time increases from 40 s (for PUR_0) to 57, 55, 62, and 51 s for PUR_WS, PUR_WS/P, PUR_WS/MMT, and PUR_WS/HL, respectively. A similar tendency is observed in the case of growth time—after the addition of walnut shell fillers, the value increases from 295 s to 358, 340, 370, and 325 s, respectively. This dependence may be connected with higher viscosity of the PUR systems containing walnut shell fillers. According to the literature, the higher viscosity of PUR systems has a significant impact on the expansion process of PUR cells and may extend the reaction time by up to several minutes [39]. Moreover, the increased viscosity influences the proper stoichiometry of the PUR synthesis reaction, slowing down the polymerization process and leading to phase separation [40]. This effect may be additionally enhanced with the presence of filler particles which contribute to limiting the mobility of the polymer chains during the PUR synthesis [46,47]. Among all studied PUR systems walnut the highest increase in the value of processing times exhibits PUR composites containing walnut shell fillers modified with montmorillonite, probably as a result of the high viscosity of such a modified PUR system. 

**Figure 4:**
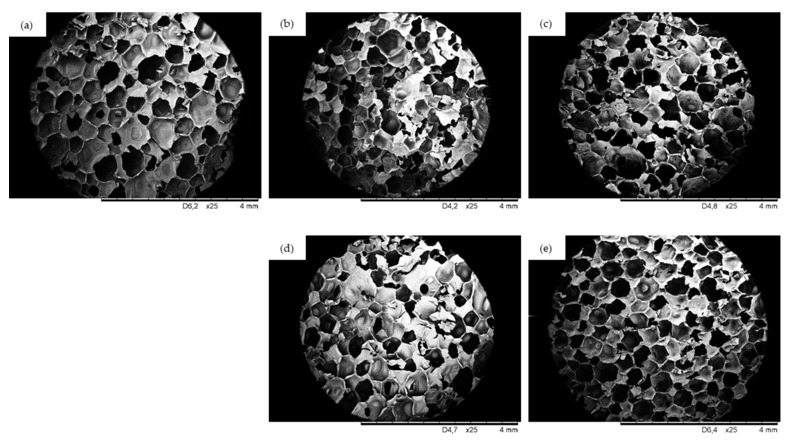
Cellular structure of (**a**) PUR_0, (**b**) PUR_WS, (**c**) PUR_WS/P, (**d**) PUR_WS/MMT, (**e**) PUR_WS/HL.

The cellular structure of PUR composites is one of the most important factors, which determines the further physico-mechanical performances of PUR composites [44,48]. Therefore, a crucial factor determining the formation of PUR composites with a well-developed closed-cell structure is the proper balance between the concentration of the filler, the viscosity of the PUR systems, and the appropriate dispersion of the filler in the PUR matrix [49]. The morphology of the reference PUR foam and PUR composites containing walnut shell fillers are presented in Figure 4.

The morphology of the reference foam is well-preserved and consists of uniform closed-cells with a negligible number of open-cells. The addition of WS filler results in a similar structure, however, the number of broken cells is slightly increased. The effect is more prominent in the case of PUR_WS/MMT. A more homogenous structure is observed in the case of PUR_WS/P and PUR_WS/HL. This may be connected with the fact that montmorillonite-modified walnut shell filler possesses larger particles, and the viscosity of such modified PUR system is higher when compared with the PUR_WS/P and PUR_WS/HL (see Figure 2). This may result in poor interfacial adhesion between the filler surface and the PUR matrix, which promotes earlier cell collapsing phenomena and increases a high possibility of generating open pores [50]. Deterioration of the foam morphology after the incorporation of the filler was reported in previous studies as well [51,52,53].

**Figure 5:**
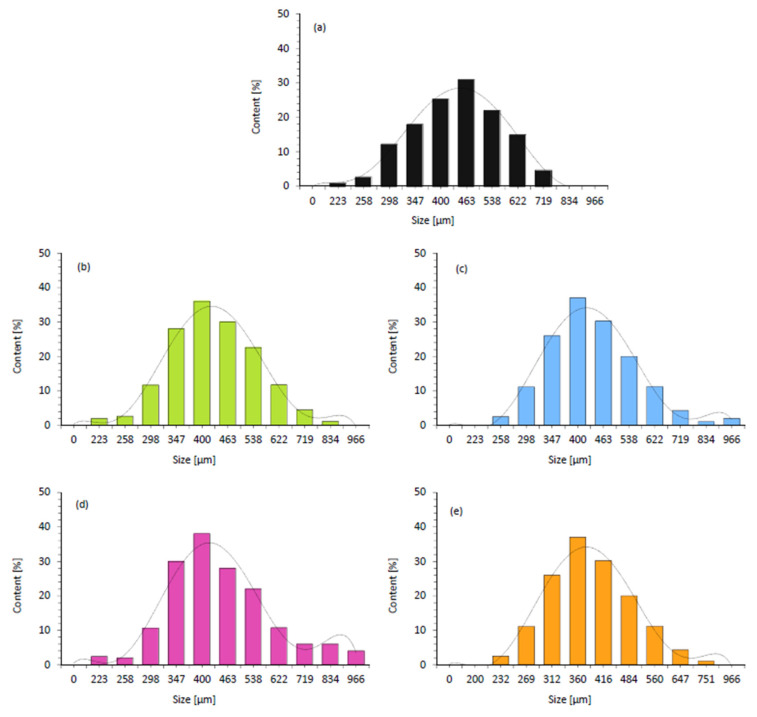
Call size distribution of (**a**) PUR_0, (**b**) PUR_WS, (**c**) PUR_WS/P, (**d**) PUR_WS/MMT and (**e**) PUR_WS/HL.

**Figure 6:**
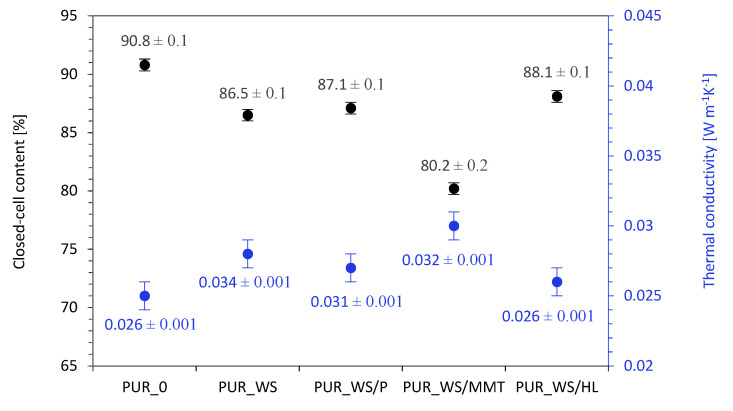
Closed-cell content and thermal conductivity results of PUR composites containing walnut shell fillers.

According to the results presented in Figure 5, the addition of walnut shell fillers affects the average cell diameter. The average cell diameter of reference foam (PUR_0) is 470 µm, and it decreases to 420, 410, 415, and 390 for PUR_WS, PUR_WS/P, PUR_WS/MMT, and PUR_WS/HL, respectively. According to the previous studies, the filler particles incorporated into the PUR system may act as nucleating centers for the formation of the cells, changing the nucleation character from homogenous to heterogonous. Because of this, greater numbers of the cells start to nucleate at the same time, which reduces the diameter of the cells. Moreover, the further expansion of the cells is limited by increased viscosity of the PUR systems, which in turn contribute to the formation of the smaller cells [54,55,56,57,58,59]. On the other hand, as presented in Figure 5. after the incorporation of the walnut shell filler, the overall distribution of the cells becomes less uniform. This effect is more prominent in the case of PUR composites containing walnut shell filler modified with montmorillonite. Previous studies have shown, that the application of the filler particles with larger diameters, results in rupturing of the cells, due to incomplete incorporation of the filler particles into the PUR matrix [50]. The confirmation of these results may be also found in the results of the closed-cell content (Figure 6). For reference foam (PUR_0) the closed-cell content is 90.8%. After the addition of walnut shell fillers, the value decreases to 86.5, 87.1, 80.2, and 88.1% for PUR_WS, PUR_WS/P. PUR_WS/MMT and PUR_WS/HL, respectively.

**Figure 7:**
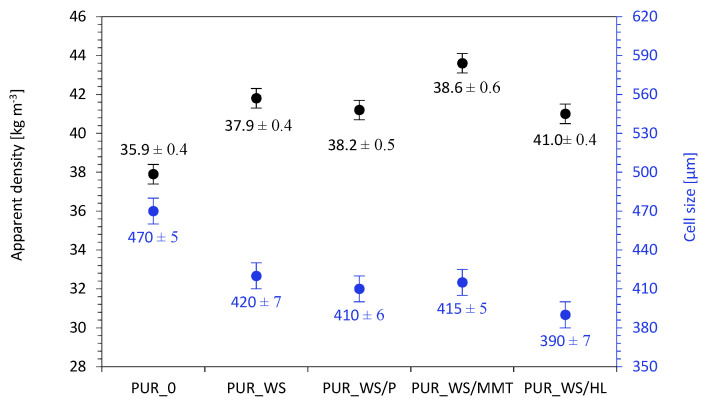
The results of apparent density and cell size of PUR composites containing walnut shell fillers.

According to the results presented in Figure 7 the addition of walnut shell fillers increases the value of apparent density of developed PUR composites. The apparent density of the reference PUR_0 is 35.9 kg m^−3^ and it increases to 37.9, 38.2, 38.6, and 41.0 kg m^−3^ for PUR_WS, PUR_WS/P, PUR_WS/MMT, and PUR_WS/HL. This is mostly connected with the increased dynamic viscosity of PUR systems containing solid particles of walnut shell fillers and the molecular weight of the fillers. However, the changes in the apparent density of PUR density are not significant and may be considered negligible. Most importantly, the addition of walnut shell fillers does not deteriorate significantly the value of thermal conductivity of PUR composites, which is a crucial factor, determining the further application of PUR materials in the construction industry. According to the result presented in Figure 6, the thermal conductivity of reference PUR_0 is 0.025 Wm^−1^ K^−1^ and it increases insignificantly to 0.028, 0.027, 0.030, and 0.026 Wm^−1^ K^−1^ for PUR_WS, PUR_WS/P, PUR_WS/MMT, and PUR_WS/HL. A slight increase in the thermal conductivity of PUR composites may be related to the presence of solid particles in the PUR matrix, which contributes to the increase in the value of λ_solid_. Among all developed PUR composites, the highest value of thermal conductivity is observed for PUR composites containing walnut shell filler modified with montmorillonite (PUR_WS/MMT). As discussed previously, such modified PUR composites are characterized by a less uniform structure with a lower content of closed-cells. Taking into consideration that the thermal conductivity of the air (λ = 0.025 Wm^−1^ K^−1^) is higher than that of the blowing agent encapsulated in the closed-cells of PUR structure (CO_2_), the thermal conductivity of PUR composites containing montmorillonite-modified walnut shell filler is increased.

The mechanical properties of polyurethane foams are dependent on the apparent density and filler properties. As shown in previous studies [27,60,61], the mechanical properties of the obtained PUR composites strongly depend on the type of filler, its surface, particle size, and its interaction with the polymer matrix. Therefore, the effect of walnut fillers on compressive strength, flexural strength, and impact strength were assessed.

**Figure 8:**
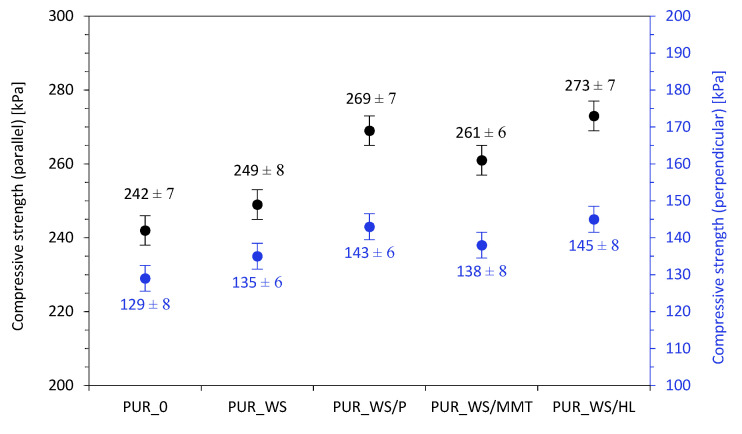
The impact of walnut fillers on the compressive strength of PUR foams.

The results of the compressive strength measured at 10% deformation (σ_10%_) are shown in Figure 8. Comparing with PUR_0 in the case of compression parallel to the foam growth direction, the value of σ_10%_ increases by ~3, 11, 8, and 13% for PUR_WS, PUR_WS/P, PUR_WS/MMT, and PUR_WS/HL, respectively. A similar relationship is observed in the case of perpendicular compression. The value of the compressive strength increases from the value of 129 kPa for PUR_0 to 135, 143, 138, and 145 kPa for PUR_WS, PUR_WS/P, PUR_WS/MMT, and PUR_WS/HL, respectively.

**Figure 9:**
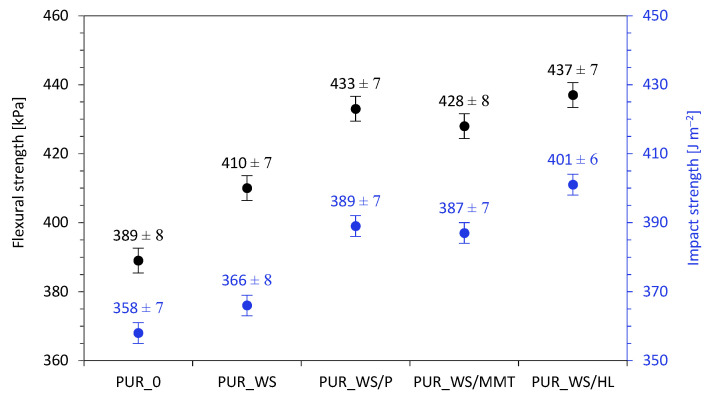
The impact of walnut shell fillers on the flexural strength and impact strength of PUR foams.

As presented in Figure 9, the incorporation of analyzed fillers influences also the flexural and impact strength of PUR composites. When comparing with PUR_0 aforementioned properties are improved. The best values for both of these properties are achieved by PUR_WS/HL. When comparing the flexural strength results with the reference foams, an increase by ~5, 11, 10, and 12% respectively for PUR_WS, PUR_WS/P, PUR_WS/MMT, and PUR_WS/HL are observed. The impact strength increases from 358 J m^−2^ for PUR_0 to 366, 389, 387 and 401 J m^−2^ for PUR_WS, PUR_WS/P, PUR_WS/MMT, and PUR_WS/HL, respectively. On the basis of the obtained results, it can be observed that the foams with the addition of walnut fillers showed improved mechanical properties. The best results were noticed for foams with the addition of modified fillers (PUR_WS/HL, PUR_WS/P, and PUR_WS/MMT). The improvement of the mechanical properties of modified foams compared to the reference foam may be related to the morphology of these foams, their viscosity, and the incorporation of reinforcing walnut filler particles into the PUR structure [57,59]. The modified foams show a better cross-linked cell structure, with smaller cells, which better stabilizes and prevents foam damage, which can be observed in better results of modified PUR foams, obtained during the analysis of flexural, compressive, and impact strength [60,61,62,63].

**Figure 10:**
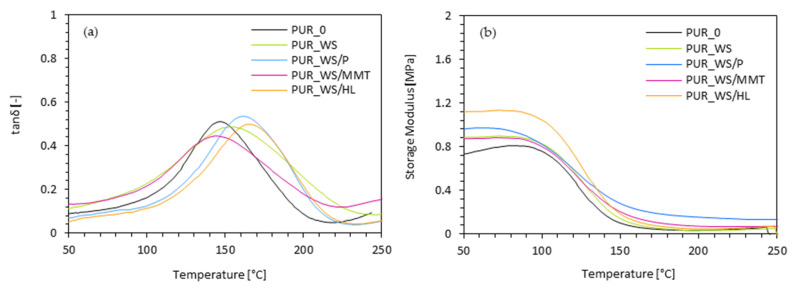
Dynamic-mechanical performance of PUR composites containing walnut shell filler–(**a**) tan δ and (**b**) storage modulus.

According to the results presented in Figure 10a, the incorporation of walnut shell fillers affects the value of T_g_. When compared with PUR_0, after the addition 2 wt.% of each filler, the values of T_g_ shift towards higher temperatures. The highest value of T_g_ exhibits PUR composites reinforced with 2 wt.% of walnut shell filler modified with halloysite–comparing to PUR_0 the value of T_g_ increases from 147 to 166 °C. These results are in agreement with the results of apparent density. As presented in Figure 8, the values of apparent density of PUR composites containing walnut shell fillers are somewhat higher, while the overall structure quite uniform with a great number of closed-cells. Some deterioration in T_g_ is observed d in the case of PUR composites reinforced with walnut shell filler modified with montmorillonite. The value of T_g_ decreases slightly to 143 °C, however, it is still comparable with the value obtained for PUR_0. This may be connected with the more porous structure of PUR_WS/MMT, which contributes to an increase in the mobility of the polymer chains. The confirmation of the T_g_ results may be also found in the results of the storage modulus (E’). According to the results presented in Figure 10b the incorporation of walnut shell fillers increases the value of E’. It is believed that well-dispersed particles of walnut shell fillers can generate some interlocks between the cells. The more complex PUR structure limits the mobility of PUR chains, thereby improving the reinforcement effect of the PUR composites.

The dimensional stability was determined based on the linear changes in dimensions, volume, and mass of PUR composites. Measurements were carried out for 14 days at +70 and −20 °C. The results obtained during the analysis are summarized in Table 2.

As can be noticed based on the presented data, the addition of the used fillers affected the dimensional stability of the foams conditioned with both higher and lower temperatures. The dimensional stability of PUR composites indicates that the addition of walnut shell fillers resulted in negligible changes in dimensional stability of the modified composites in relation to the reference foam. However, when comparing the results obtained during the test, a decrease in changes in width, length, and thickness is observed. This proves that the addition of fillers minimally but increases the dimensional stability of the modified PUR composites.

The burning behavior of PUR composites was assessed using the cone calorimetry method. Table 3 presents summarized results obtained during the experiment including the ignition time (IT), the peak rate of heat release (pHRR), the total heat release (THR), the total smoke release (TSR), the average yield of CO, and CO_2_ (COY and CO_2_Y), and the limiting oxygen index (LOI).

**Figure 11:**
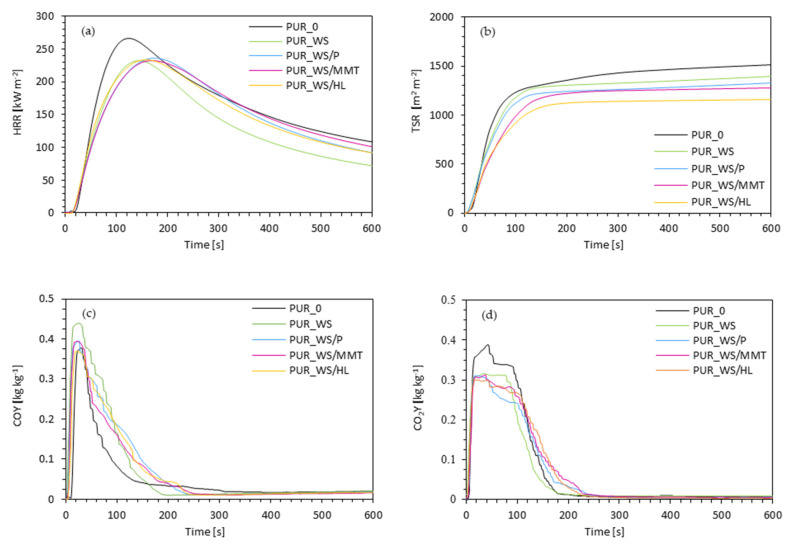
The results of the cone calorimeter experiment—(**a**) the peak rate of heat release (pHRR), (**b**) the total smoke release (TSR), (**c**) the average yield of CO, and (**d**) the average yield of CO_2_.

When comparing the modified PUR composites to the reference PUR_0, it can be observed that the modifications affected the ignition time (IT) slightly. The ignition time increases from 4 s for PUR_0 to 6 s for PUR_WS, 7 s for PUR_WS/MMT, and 8 s for PUR_WS/P and PUR_WS/HL, respectively. The intensity of the flame was determined by the heat peak release value (pHRR), related to the release of low molecular weight compounds (olefins, amines, or isocyanates). As it can be noticed in Figure 11, all analyzed PUR composites show one main peak of this parameter. When compared with PUR_0, which has a peak value of 265 kW m^−2^, PUR composites show lower values of this parameter and are 236, 234, 233, and 232 kW m^−2^ respectively for PUR_WS/P, PUR_WS/HL, PUR_WS/MMT, and PUR_WS, which proves a slight reduction of heat released during combustion of PUR composites. The incorporation of walnut fillers affects also the total heat release (THR). The value of this parameter increased for PUR_WS to 23.8 MJ m^−2^, comparing with PUR_0 (21.9 MJ m^−2^), and decreased for PUR_WS/MMT (21.3 MJ m^−2^), PUR_WS/P (20.7 MJ m^−2^), and PUR_WS/HL (20.1 MJ m^−2^).

When analyzing the effect of modifiers on the total smoke release (TSR) presented in Figure 11, it can be noticed that the addition of walnut fillers decreased this value from 1516 m^2^ m^−2^ for the reference foam to 1394, 1329, 1274, and 1160 m^2^ m^−2^ for PUR_WS, PUR_WS/P, PUR_WS/MMT, and PUR_WS/HL, respectively. The inclusion of walnut fillers also affected the amount of gas released. Compared to the foam PUR_0, the amount of carbon monoxide released increased from 0.375 kg kg^−1^ to 0.393 and 0.394 kg kg^−1^ in the case of PUR_WS/P and PUR_WS/MMT, and to even 0.439 kg kg^−1^ for PUR_WS and decreased to 0.368 kg kg^−1^ in the case of PUR_WS/HL. On the other hand, in the case of carbon dioxide, all modified foams showed a lower amount of released gas. The CO_2_Y parameter was 0.388 kg kg^−1^ for the reference foam and decreased to 0.315, 0.312, 0.311 and even 0.299 kg kg^−1^ for PUR_WS, PUR_WS/MMT, PUR_WS/P and PUR_WS/HL, respectively. Concerning the limiting oxygen index (LOI), it could be seen that the used fillers also influenced this parameter. The PUR_WS (19.9%) showed a slightly lower LOI value than the reference foam (20.2%). On the other hand, the remaining foams achieved better, higher values of this parameter. Compared to PUR_0, the LOI value increased to 20.8% for PUR_WS/MMT, 21.0% for PUR_WS/P, and PUR_WS/HL—reaching the highest value of 21.5%. Based on the obtained results, it could be concluded that the application of modified walnut filler can improve the flammability properties of PUR composites. Among the analyzed composites, the foam PUR_WS/HL showed the best fire resistance properties in this experiment.

**Figure 12:**
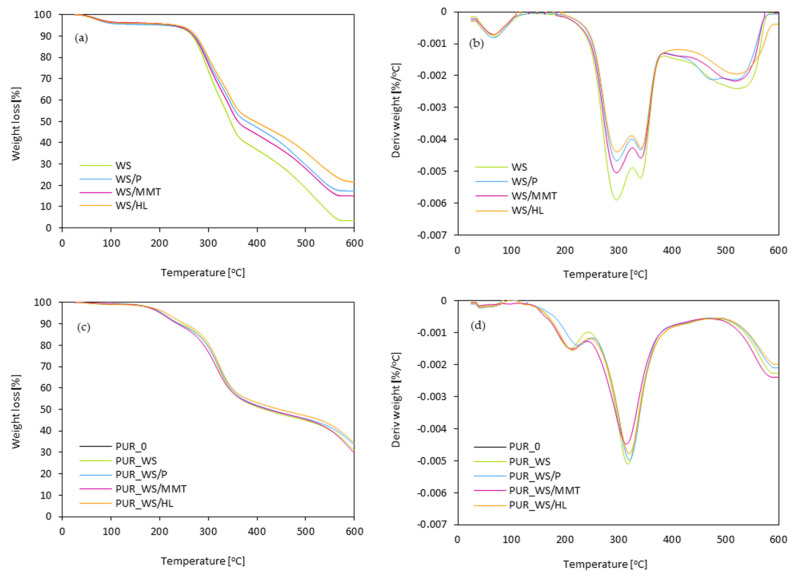
Thermogravimetric (TGA) and derivative thermogravimetry (DTG) results of (**a**,**b**) walnut shell fillers and (**c**,**d**) PUR composites.

To assess the effect of walnut shell fillers on the thermal stability of the PUR composites the thermogravimetric analysis (TGA) and derivative thermogravimetry analysis (DTG) were performed. During the study, the following stages of thermal decomposition were determined. Moreover, char residues at the temperature of 600 °C were determined. The results of TGA/DTG analysis are presented in Figure 12 and Table 4. According to the results presented in Figure 12a,b, it can be concluded, that in the case of non-modified walnut shell filler, three stages of thermal decompositions are observed. The first stage of mass loss occurs at a relatively low temperature (~100 °C) and refers to the evaporation of the moisture absorbed by the filler and volatile compounds (low molecular weight esters and fatty acids) which are inherent to the filler. The second stage representing 30% of mass loss occurs between 300 and 500 °C. The maximum rate at ~300 °C refers to the thermal degradation of cellulose and hemicellulose [64,65]. The last step of thermal decomposition occurs between 400 and 500 °C with the maximum rate at ~460 °C and refers to the thermal decomposition of lignin [65]. When comparing with non-modified walnut shell filler, the walnut shell fillers modified with perlite, montmorillonite, and halloysite also reveal three main stages of thermal decomposition; however, the weight loss of hemicellulose and lignin is reduced. This behavior may be connected with the presence of thermal protective layers created by layered fillers—perlite, montmorillonite, and halloysite, which effectively improve the thermal stability of modified walnut shell fillers.

DSC/TGA analysis of PUR composites is presented in Figure 12c,d. In general, thermal degradation of PUR porous materials involves three stages. The individual stages correspond to characteristic temperatures (T_max_) determined based on the results obtained during thermogravimetric analysis (TGA). The first stage of thermal degradation (T_max1_) is related to the thermal decomposition of low molecular weight compounds [66,67]. In the case of analyzed PUR composites, the first stage of decomposition occurs between 203 and 221 °C. It can be noticed that the incorporation of walnut shell fillers results in higher values of T_max1_, which can be related to the modification of the filler with natural flame retardant compounds—perlite, montmorillonite, and halloysite [68]. The value of T_max1_ increases from 209 °C (for PUR_0) to 221, 209, and 211 °C, respectively. The slight deterioration in thermal stability of PUR composites is observed after the incorporation of unmodified walnut shell filler—the value of T_max1_ decreases slightly from 209 to 203 °C due to the cellulosic nature of the filler. The second stage of thermal degradation refers to the thermal degradation of hard segments of PUR composites and thermal degradation of walnut shell fillers (cellulose/hemicellulose/lignin) [60]. In all cases, the thermal degradation occurs in the range between 306 and 325 °C. Based on the obtained results, it can be concluded that the greatest improvement in thermal degradation of PUR composites is observed for PUR composites containing walnut shell filler modified with halloysite—the value of T_max2_ increases from 306 to 325 °C. The last, third stage of thermal degradation (T_max3_) is related to the degradation of the compounds and fragments that were generated during previous stages [69]. In the case of analyzed PUR composites, it occurs in the temperature range between 571 and 599 °C. The incorporation of the walnut shell fillers results in a slight increase in the temperature characteristic for this stage T_max3_. The greatest improvement in T_max3_ is observed for PUR composites containing walnut shell filler modified with halloysite compound. By analyzing the influence of walnut shell fillers on the thermal stability of the obtained PUR composites, the char residue amount at 600 °C was also evaluated. When comparing the carbonization residue with the PUR_0 result, it can be concluded that with increasing walnut shell fillers content, the residue content also increases. More specifically, the amount of char residue increases from 22.6% for PUR_0 to 31.8, 35.0, 31.3 and 35.8%, respectively for PUR_WS, PUR_WS/P, PUR_WS/MMT and PUR_WS/HL. Based on the obtained results, it can be concluded that the incorporation of walnut shell fillers improves the thermal stability of the obtained PUR composites.

## 3. Materials and Methods

### 3.1. Materials

Polymeric diphenulmethane diisocyanate with the brand name of Purocyn B was supplied by Purinova Company (Bydgoszcz, Poland). Polyether polyol with the brand name of Stepanpol PS-2352 was purchased from Stepan Company (Northfield, IL, USA). Potassium octoate with a brand name of Kosmos 75 and potassium acetate with a brand name of Kosmos 33 was supplied by Evonik Industry (Essen, Germany). The silicone-based surfactant with the brand name Tegostab B8513 was purchased from Evonik Industry (Essen, Germany). Pentane and cyclopentane (used as a blowing agent in a volume ratio of 50:50 *v*/*v*) were supplied by Sigma-Aldrich Corporation (Saint Louis, MO, USA). Walnut shells (WS) were supplied by a local company from Poland. The halloysite powder with a specific surface of 64 m^2^/g, material was purchased from by Sigma-Aldrich (Saint Louis, MO, USA), montmorillonite clay, Nanomer^®^ contains 15–35 wt. % octadecylamine, 0.5–5 wt.% aminopropyltriethoxysilane was purchased from Merck (Darmstadt, Germany), perlite powder with a specific surface of 60–70 m^2^/g was supplied by Sigma-Aldrich (Saint Louis, MO, USA).

### 3.2. Synthesis of PUR Composites 

**Figure 13:**
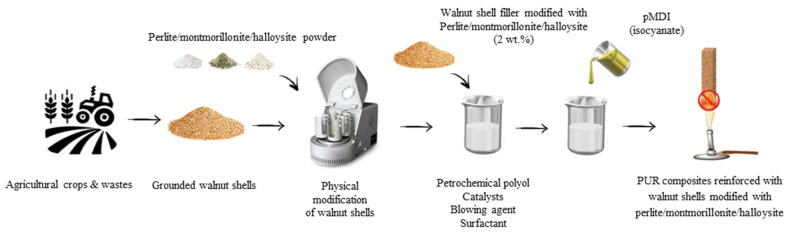
Schematic procedure of the synthesis of PUR composites reinforced with walnut shells modified with perlite/montmorillonite/halloysite.

Before adding to the polyol system, walnut shell filler was mechanically ground and sieved using a 100 µm sieve. In the next step, walnut shell filler was modified with perlite/montmorillonite/halloysite using a high-energy ball milling process. Walnut shell filler was mixed with perlite/montmorillonite/halloysite powder (walnut shell filler weight to perlite/montmorillonite/halloysite weight ratio = 1:2) and milled using a high-energy ball milling process (60 min, 3000 rpm, ball weight to powder weight ratio = 12:1). Walnut shells modified with perlite/montmorillonite/halloysite were used as reinforcing filler in the synthesis of PUR composites. The impact of the type of filler on the selected physical and mechanical properties of PUR composites was determined. Each component of the PUR system was added based on the weight percentage of polyol (Stepanpol PS2352). The preweighted amount of polyol, blowing agent (pentane/cyclopentane), surfactant (Tegostab B8513), catalysts (Kosmos 33, Kosmos 75), and water were placed in the beaker and mechanically stirred for 60 s (2000 RPM). In the next step, the walnut shell filler modified with perlite/montmorillonite/halloysite was added to the mixture and continually stirred for another 60 s. After that, the polymeric diphenylmethane diisocyanate (Purocyn B) was added to the mixture and such obtained system was mixed vigorously for 30 s (2000 RPM). As synthesized PUR composites were freely expanded and cured for 24 h at room temperature. The produced PUR composites were labeled as PUR_0, PUR_WS, PUR_WS/P, PUR_WS/MMT, and PUR_WS/HL. The formulations of PUR composites reinforced with walnut shell fillers are given in Table 5. The schematic procedure of the synthesis of PUR composites presents Figure 13.

### 3.3. Methods and Instruments

Morphology and cell size distribution of foams were determined based on the cellular structure pictures of foams taken using JEOL JSM-5500 LV scanning electron microscopy (JEOL LTD, Akishima, Japan). The average pore diameters and pore size distribution were determined using ImageJ software (Media Cybernetics Inc. Rockville, MD, USA). The closed-cell content was determined according to PN-EN ISO 4590 using the helium pycnometer AccuPyc 1340 with the FoamPyc option (Micrometrics, Norcross, GA, USA) in S.Z.T.K. ‘TAPS’—Maciej Kowalski Company (Lodz, Poland). The chemical structure of fillers was determined by Fourier-transform infrared spectroscopy (FTIR, Nicolet iS50 spectrometer, Thermo Fisher Scientific, Waltham, MA, USA). The average size of filler particles was assessed with the dynamic light scattering (DLS) method, using a Zetasizer NanoS90 instrument (Malvern Instruments Ltd., Malvern, UK). The apparent density of foams was examined accordingly to the standard ASTM D1622, which is equivalent to ISO 845. A three-point bending test of foams was carried out following the standard ASTM D7264, which is equivalent to ISO 178, using Zwick Z100 Testing Machine (Zwick/Roell Group, Ulm, Germany). The compressive strength (σ_10%_) of foams was examined accordingly to the standard ASTM D1621, which is equivalent to ISO 844, using Zwick Z100 Testing Machine (Zwick/Roell Group, Ulm, Germany). The impact examination was carried out accordingly to the standard ASTM D4812, which is equivalent to ISO 180, using Zwick Z100 Testing Machine (Zwick/Roell Group, Ulm, Germany). The dimensional stability of foams was determined accordingly to the standard ASTM D2126, which is equivalent to ISO 2796. The thermal stability of foams was determined using a Mettler Toledo thermogravimetric analyzer TGA/DSC1 (Columbus, OH, USA). The burning behavior of foams was determined accordingly to the standard ISO 5660 using the cone calorimeter apparatus in S.Z.T.K ‘TAPS’—Maciej Kowalski Company (Saugus, Poland). The thermal conductivity of the foams was determined using the LaserComp 50 heat flow meter (HFMA, Westchester, IL, USA).

## 4. Conclusions

Polyurethane (PUR) composites were modified with 2 wt.% of walnut shell filler modified with selected mineral filler—perlite, montmorillonite, and halloysite. The impact of modified walnut shell fillers on selected properties of PUR composites, such as rheological properties (dynamic viscosity, foaming behavior), mechanical properties (compressive strength, flexural strength, impact strength), dynamic-mechanical behavior (glass transition temperature, storage modulus), insulation properties (thermal conductivity), thermal characteristic (temperature of thermal decomposition stages), and flame retardant properties (e.g., ignition time, limiting oxygen index, heat peak release) was investigated. Among all modified types of PUR composites, the greatest improvement was observed for PUR composites filled with walnut shell filler functionalized with halloysite. For example, on the addition of such modified walnut shell filler, the compressive strength was enhanced by ~13%, flexural strength by ~12%, and impact strength by ~14%. Due to the functionalization of walnut shell filler with thermally stable flame retardant compounds, such modified PUR composites were characterized by higher temperatures of thermal decomposition. Most importantly, PUR composites filled with flame retardant compounds exhibited improved flame resistance characteristics—in all cases, the value of peak heat release was reduced by ~12%, while the value of total smoke release was reduced by ~23%. Moreover, the incorporation of walnut shell fillers modified with perlite, montmorillonite, and halloysite improved the thermal stability of PUR composites—e.g., the value of char residue (measured at 600 °C) increased from 22.6% to 35.8%, for PUR composites containing halloysite. 

## Figures and Tables

**Table 1:** Processing times of PUR mixtures containing walnut shell fillers.

Sample	Processing Times [s]		
	Start Time	Growth Time	Tack-Free Time
PUR_0	40 ± 2	295 ± 6	358 ± 8
PUR_WS	57 ± 3	358 ± 5	346 ± 9
PUR_WS/P	55 ± 2	340 ± 7	348 ± 7
PUR_WS/MMT	62 ± 3	370 ± 8	340 ± 8
PUR_WS/HL	51 ± 3	325 ± 6	355 ± 7

**Table 2:** Dimensional stability of PUR composites containing walnut shell fillers.

Sample	Dimensional Stability at 70 °C [%]			Dimensional Stability at −20 °C [%]		
	Width	Length	Thickness	Width	Length	Thickness
PUR_0	1.82 ± 0.01	1.70 ± 0.01	1.85 ± 0.01	1.93 ± 0.01	1.79 ± 0.01	1.77 ± 0.01
PUR_WS	1.72 ± 0.01	1.64 ± 0.01	1.77 ± 0.01	1.84 ± 0.01	1.77 ± 0.01	1.73 ± 0.01
PUR_WS/P	1.68 ± 0.01	1.53 ± 0.01	1.73 ± 0.01	1.76 ± 0.01	1.69 ± 0.01	1.65 ± 0.01
PUR_WS/MMT	1.66 ± 0.01	1.42 ± 0.01	1.67 ± 0.01	1.73 ± 0.01	1.74 ± 0.01	1.71 ± 0.01
PUR_WS/HL	1.65 ± 0.01	1.49 ± 0.01	1.71 ± 0.01	1.77 ± 0.01	1.77 ± 0.01	1.68 ± 0.01

**Table 3:** The results of the cone calorimeter experiment.

Sample	IT (s)	pHRR (kW m^−2^)	THR (MJ m^−2^)	TSR (m^2^ m^−2^)	COY (kg kg^−1^)	CO_2_Y (kg kg^−1^)	LOI (%)
PUR_0	4	265	21.9	1516	0.375	0.388	20.2
PUR_WS	6	232	23.8	1394	0.439	0.315	19.9
PUR_WS/P	8	236	20.7	1329	0.393	0.311	21.0
PUR_WS/MMT	7	233	21.3	1274	0.394	0.312	20.8
PUR_WS/HL	8	234	20.1	1160	0.368	0.299	21.5

**Table 4:** The results of thermal stability of PUR composites.

Sample	T_max_ (°C)			Char Residue (wt. %) at 600 °C
	1st Stage	2nd Stage	3rd Stage	
PUR_0	209 ± 4	306 ± 4	571 ± 4	22.6 ± 0.2
PUR_WS	203 ± 5	319 ± 5	583 ± 5	31.8 ± 0.1
PUR_WS/P	221 ± 2	321 ± 4	595 ± 5	35.0 ± 0.2
PUR_WS/MMT	209 ± 2	313 ± 2	590 ± 2	31.3 ± 0.2
PUR_WS/HL	211 ± 5	325 ± 4	599 ± 6	35.8 ± 0.2

**Table 5:** The formulations of PUR composites reinforced with walnut shell fillers.

Component	PUR_0	PUR_WS	PUR_WS/P	PUR_WS/MMT	PUR_WS/HL
	Parts by Weight (wt.%)				
Stepanpol PS-2352	100				
Purocyn B	160				
Kosmos 75	6				
Kosmos 33	0.8				
Tegostab B8513	2.5				
Water	0.5				
Pentane/cyclopentane	11				
Walnut shells	0	2	0	0	0
Walnut shells modified with perlite	0	0	2	0	0
Walnut shells modified with montmorillonite	0	0	0	2	0
Walnut shells modified with halloysite	0	0	0	0	2

## Data Availability

No data available.
